# EasyAmplicon: An easy‐to‐use, open‐source, reproducible, and community‐based pipeline for amplicon data analysis in microbiome research

**DOI:** 10.1002/imt2.83

**Published:** 2023-01-27

**Authors:** Yong‐Xin Liu, Lei Chen, Tengfei Ma, Xiaofang Li, Maosheng Zheng, Xin Zhou, Liang Chen, Xubo Qian, Jiao Xi, Hongye Lu, Huiluo Cao, Xiaoya Ma, Bian Bian, Pengfan Zhang, Jiqiu Wu, Ren‐You Gan, Baolei Jia, Linyang Sun, Zhicheng Ju, Yunyun Gao, Tao Wen, Tong Chen

**Affiliations:** ^1^ Shenzhen Branch, Guangdong Laboratory of Lingnan Modern Agriculture, Genome Analysis Laboratory of the Ministry of Agriculture and Rural Affairs, Agricultural Genomics Institute at Shenzhen Chinese Academy of Agricultural Sciences Shenzhen Guangdong China; ^2^ Department of Vascular Surgery, Fu Xing Hospital Capital Medical University Beijing China; ^3^ State Key Laboratory of Grassland Agro‐ecosystems, Centre for Grassland Microbiome, College of Pastoral Agricultural Science and Technology Lanzhou University Lanzhou Gansu China; ^4^ Centre for Agricultural Resources Research, Institute of Genetics and Developmental Biology Chinese Academy of Sciences Shijiazhuang China; ^5^ College of Environmental Science and Engineering North China Electric Power University Beijing China; ^6^ Institute of Microbiology Chinese Academy of Sciences Beijing China; ^7^ Department of Pediatrics, Affiliated Jinhua Hospital Zhejiang University School of Medicine Jinhua Zhejiang China; ^8^ College of Natural Resources and Environment Northwest A&F University Yangling Shaanxi China; ^9^ Key Laboratory of Oral Biomedical Research of Zhejiang Province, Cancer Center of Zhejiang University, Clinical Research Center for Oral Diseases of Zhejiang Province, School of Stomatology, Zhejiang University School of Medicine Stomatology Hospital Hangzhou Zhejiang China; ^10^ Department of Microbiology University of Hong Kong Hong Kong China; ^11^ Center of Excellence in Fungal Research Mae Fah Luang University Chiang Rai Thailand; ^12^ Graduate School of Frontier Sciences University of Tokyo Chiba Japan; ^13^ Department of Plant‐Microbe Interactions Max Planck Institute for Plant Breeding Research Cologne Germany; ^14^ APC Microbiome Institute University College Cork Cork Ireland; ^15^ Department of Genetics, University Medical Center Groningen University of Groningen Groningen The Netherlands; ^16^ Singapore Institute of Food and Biotechnology Innovation (SIFBI), Agency for Science Technology and Research (A*STAR) Singapore Singapore; ^17^ Department of Life Science Chung‐Ang University Seoul Republic of Korea; ^18^ The Key Laboratory of Plant Immunity Jiangsu Provincial Key Lab for Organic Solid Waste Utilization Jiangsu Collaborative Innovation Center for Solid Organic Waste Resource Utilization, National Engineering Research Center for Organic‐Based Fertilizers Nanjing Agricultural University Nanjing China; ^19^ National Resource Center for Chinese Materia Medica China Academy of Chinese Medical Sciences Beijing China

**Keywords:** amplicon, bioinformatics, microbiome, pipeline, visualization, metagenome

## Abstract

It is difficult for beginners to learn and use amplicon analysis software because there are so many software tools to choose from, and all of them need multiple steps of operation. Herein, we provide a cross‐platform, open‐source, and community‐supported analysis pipeline EasyAmplicon. EasyAmplicon has most of the modules needed for an amplicon analysis, including data quality control, merging of paired‐end reads, dereplication, clustering or denoising, chimera detection, generation of feature tables, taxonomic diversity analysis, compositional analysis, biomarker discovery, and publication‐quality visualization. EasyAmplicon includes more than 30 cross‐platform modules and R packages commonly used in the field. All steps of the pipeline are integrated into RStudio, which reduces learning costs, keeps the flexibility of the analysis process, and facilitates personalized analysis. The pipeline is maintained and updated by the authors and editors of WeChat official account “Meta‐genome.” Our team will regularly release the latest tutorials both in Chinese and English, read the feedback from users, and provide help to them in the WeChat account and GitHub. The pipeline can be deployed on various platforms, and the installation time is less than half an hour. On an ordinary laptop, the whole analysis process for dozens of samples can be completed within 3 h. The pipeline is available at GitHub (https://github.com/YongxinLiu/EasyAmplicon) and Gitee (https://gitee.com/YongxinLiu/EasyAmplicon).

## Highlights

- EasyAmplicon is a user‐friendly, cross‐platform, and community‐supported pipeline for amplicon data analysis.
- It has most of the modules for data processing and visualization in microbiome research.
- The pipeline is maintained and updated regularly, and we encourage users to contribute appropriate code.

## Graphical abstract

EasyAmplicon is a user‐friendly, cross‐platform, and community‐supported pipeline for amplicon data analysis. It has most of the modules for data processing and visualization in microbiome research. The pipeline is maintained and updated regularly. We encourage users to contribute appropriate code.

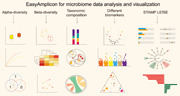

## INTRODUCTION

The rapid development of high‐throughput sequencing technologies in the past 20 years has promoted an increasingly deeper exploration of the crucial roles of microbiome in humans [1–5], animals [6–8], plants [9–11], and the environment [12–14]. Most of them were driven by amplicon sequencing (such as 16S rDNA sequencing of bacteria or archaea, eukaryotic 18S rDNA or internal transcribed spacer, and nitrogen‐fixing prokaryote's *nifH* gene) and had profiled the taxonomic composition of the microbiome in various environments [15–17].

Wet laboratory operations of amplicon sequencing are now standardized, and most operations are implemented by specialized biotechnology companies or sequencing centers. However, bioinformatics analyses of amplicon data are still challenging, and the existence of overwhelming software, methods, and algorithms brings difficult choices for beginners. The popular amplicon analysis pipelines include mothur [18], USEARCH [19], and QIIME [20], all of which have been cited over 10,000 times. However, they still have obvious shortcomings, such as a lack of downstream statistical analyses and visualization solutions, higher time costs, and being limited to specified operating systems. Some online analysis webservers are easy to use, such as Qiita [21], MGnify [22], and gcMeta [23], but they also have several limitations, such as slow upload speed, long waiting/running time, and few adjustable parameters, which make it impossible to conduct customized analyses [24, 25].

The lack of an easy‐to‐use and flexible amplicon analysis pipeline seriously restricts researchers to understand the data analysis process and hinders the development of this field. Therefore, we developed an easy‐to‐use, open‐source, and cross‐platform amplicon analysis pipeline—EasyAmplicon. It can be used in both command‐line mode and interactive mode in RStudio. Currently, it provides more than 20 visualization styles and generates publication‐quality figures easily. The open‐source code could facilitate reproducible analysis and allow personalized modification. In addition, it also generates standard input for the most popular software, such as STAMP [26], LEfSe [27], PICRUSt 1 & 2 [28, 29], BugBase [30], FAPROTAX [31], ImageGP[32], and iTOL [33]. EasyAmplicon provides a free, reproducible, and personalized solution for amplicon analysis, which could be an amazing software tool for microbiome research.

## RESULTS

### Overview of EasyAmplicon pipeline

EasyAmplicon is an integrated pipeline for amplicon data analysis and visualization on a laptop or server, and it provides various tables and figures to explore underlying biological interpretations. This pipeline is easy to install on Windows, MacOS, and Linux systems. The installation method is described in detail in the Methods section or available at https://github.com/YongxinLiu/EasyAmplicon. For the test data, comprising 18 samples and 50,000 PE250 reads per sample, the complete analysis could be finished within about 3 h with a peak memory footprint of less than 4 gigabytes (CPU: 2 cores, 2.1 GHz).

**Figure 1:**
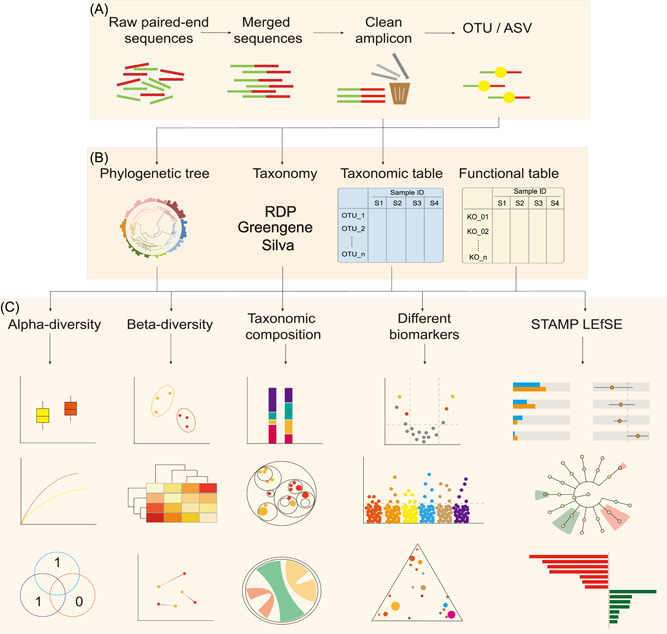
Pipeline of EasyAmplicon for analyzing paired‐end amplicon sequences. (A) Dimensionality reduction: processing raw sequencing reads into feature tables. (B) Analysis: providing phylogenetic analysis, taxonomic classification, functional prediction, and alpha‐ and beta‐diversity calculations. (C) Statistics and visualization: generating publication‐quality figures and performing statistical tests for biological interpretations. ASVs, amplicon sequence variants; OTU, operational taxonomic units.

The EasyAmplicon is an end‐to‐end pipeline. It starts with raw reads and ends with data tables and publication‐quality figures (Figure 1). It mainly comprises three steps: dimensionality reduction, analysis, and visualization & statistics (Figure 1). All the related software is easy to install (Table 1), and we provide a batch download package to accelerate pipeline deployment.

**Table 1:** Software and packages included in EasyAmplicon

Software	Function in the pipeline	Website
Git for Windows	Provides Linux Shell like environment in Windows	http://gitforwindows.org/
R	Statistical processing and data visualization	https://www.r-project.org
RStudio	Integrated development environment for R and Shell	https://posit.co/
VSEARCH	A fast, free, and cross‐platform pipeline for amplicon sequencing analysis [34]	https://github.com/torognes/vsearch
USEARCH	Processes sequences and calculates alpha‐ and beta‐diversities [19]	http://www.drive5.com/usearch/
SeqKit	Toolkit for FASTA or FASTQ file manipulation [35]	https://github.com/shenwei356/seqkit
ggplot2	R package for data visualization	https://github.com/tidyverse/ggplot2
ggClusterNet	R package for microbiome network visualization [36]	https://github.com/taowenmicro/ggClusterNet/
vegan	R package for alpha and beta diversity analysis	https://cran.r-project.org/package=vegan
ggraph	Creates layout for tree map and circle packing chart	https://github.com/thomasp85/ggraph
circlize	Circular visualization [37]	https://github.com/jokergoo/circlize

### Running the pipeline in command line or R markdown mode

**Figure 2:**
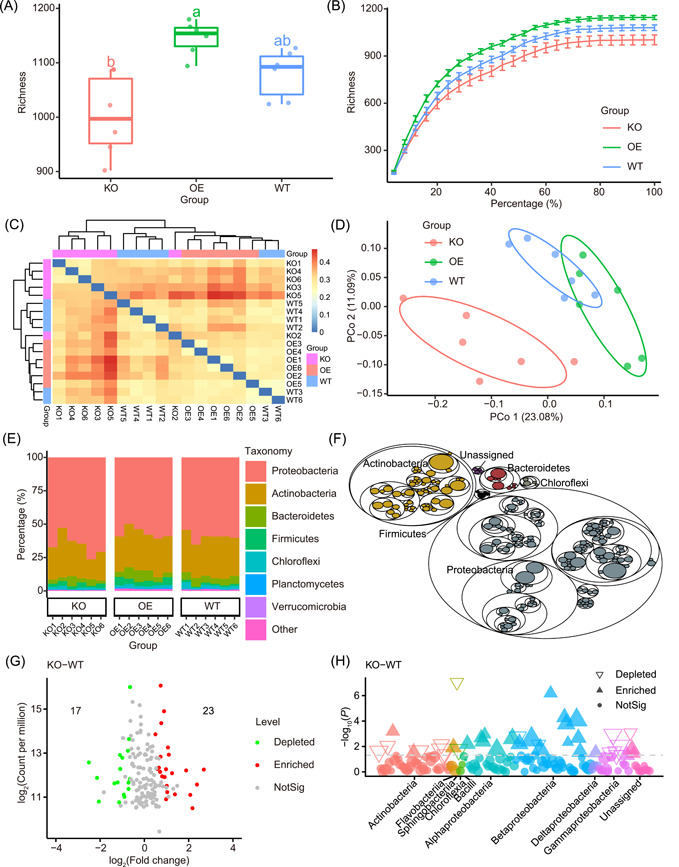
Examples of publication‐quality visualizations. (A) Boxplot showing alpha diversity in richness metrics among groups. Different letters indicate statistical significance among groups (*p* < 0.05, ANOVA, Tukey HSD). The horizontal bars within boxes represent medians. The tops and bottoms of the boxes represent the 75th and 25th percentiles, respectively. The upper and lower whiskers extend to data no more than 1.5× the interquartile range from the upper and lower edge of the box, respectively. (B) Rarefaction curve of richness shows that features reach saturation stage with increasing sequencing depth. Each vertical bar represents standard error. (C) Heatmap based on Bray−Curtis dissimilarity. (D) Principal coordinate analysis (PCoA) of Bray−Curtis dissimilarity. (E) Stacked bar plot of taxonomic composition in grouped samples at phylum level. (F) Tree map of taxonomic composition. (G) Volcano plot showing significantly differential abundance taxa between KO and WT groups. (H) Manhattan plot showing different features and related taxa between KO and WT groups. The numbers of replicated samples in this figure are as follows: in KO (*n* = 6), OE (*n* = 6), and WT (*n* = 6). KO, knock‐out; OE, overexpression; WT, wild‐type.

**Figure 3:**
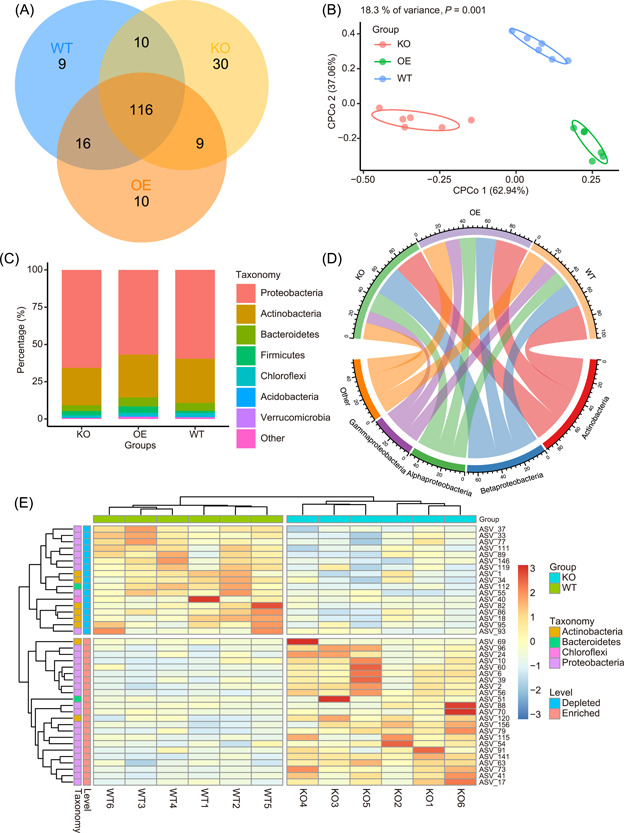
Supplementary examples of publication‐quality visualizations to Figure 2. (A) Venn diagram showing common and unique ASVs (relative abundance >0.1%) among three groups. (B) Constrained principal coordinate analysis (CPCoA) of three groups. (C) Stacked plot of average relative abundance at phylum level of three groups. (D) Circle plot of average relative abundance at phylum level of three groups. (E) Heatmap showing significantly different ASVs between KO and WT groups (Wilcoxon test, *p* < 0.05). ASVs, amplicon sequence variants; KO, knock‐out; WT, wild‐type.

First, we open the pipeline file “pipeline.sh” using RStudio. After setting the working directory, the analysis process could be run step‐by‐step with only a mouse clicking the “Run” button. For users to conduct their own analysis, only the raw sequencing data and sample metadata are needed, and the following analysis would be processed by EasyAmplicon. If RStudio is not applicable, we can copy and paste the scripts into the pipeline.sh and run them in any Shell environment (such as a terminal in Linux/Mac either locally or remotely, or Git bash in Windows). All the related software and packages are listed in Table 1. All the figures are saved in PDF format by default, and some examples are shown in Figures 2 and 3.

To make statistics and visualization of microbiome data more personalized, users can open the “Tutorial.Rmd” document in RStudio and then modify the details of the figures, such as item order, color scheme, legend layout, and so on. It can even generate a publish‐ready combo figure (Figures 2 and 3) and a reproducible HTML format report (Tutorial.html).

### Third‐party software supporting

**Figure 4:**
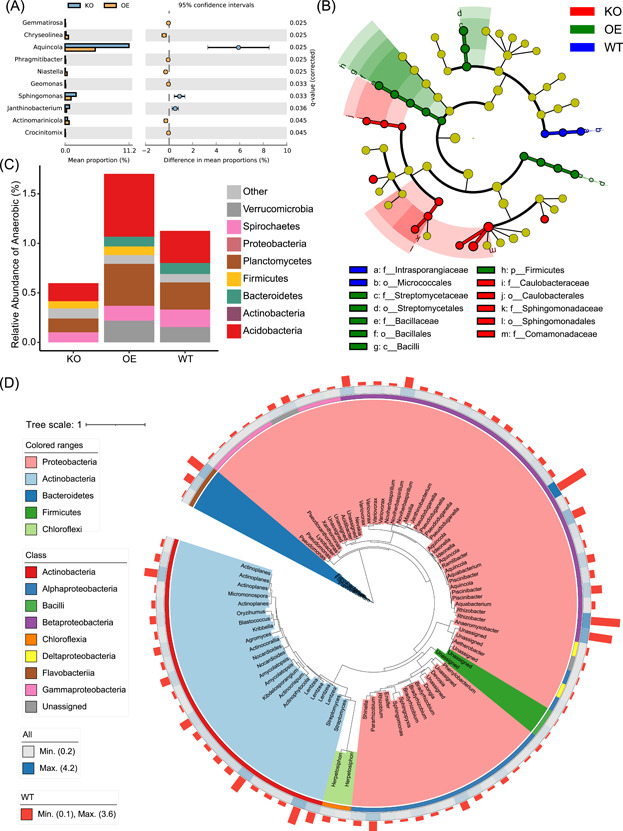
Visualizations generated by third‐party software using the intermediate files of EasyAmplicon. (A) Extended error bar plot at genus level in WT and KO groups by STAMP. (B) Cladogram showing biomarkers in each group by LEfSe. (C) Percentage of BugBase annotated anaerobic bacteria at the phylum level. (D) Phylogenetic tree of 86 ASVs (relative abundance > 0.2%). The tree background is colored by Phylum. The outer strip represents different classes. The heatmap represents the average relative abundance of all samples. The bar plot represents the relative abundance of the WT group. ASVs, amplicon sequence variants; KO, knock‐out; WT, wild‐type.

EasyAmplicon does not cover all the functions required for microbiome analysis. There are currently some mainstream and very distinctive microbiome analysis tools, such as STAMP [26], LEfSe [27], PICRUSt 1 & 2 [28, 29], BugBase [30], FAPROTAX [31], and iTOL [33]. However, some input files are difficult to prepare for users without bioinformatics backgrounds. In EasyAmplicon, a lot of scripts are used to prepare input for all the above software tools. The example visualizations, STAMP (Figure 4A), LEfSe (Figure 4B), BugBase (Figure 4C), and iTOL (Figure 4D) are shown. As for the most popular QIIME 2 pipeline, the intermediate files generated by EasyAmplicon can be imported into QIIME 2, and the output files from QIIME 2 can also be imported into EasyAmplicon for downstream analyses.

### Anticipated results

EasyAmplicon provides multiple visualization styles for amplicon data analysis. For alpha diversity (within‐sample diversity), the boxplot is the best way to visualize the data and compare each group (Figure 2A), and the different letters represent significant differences (*p* < 0.05, ANOVA, Tukey HSD test). Rarefaction analysis reveals that the features reach the saturation stage with increasing sequencing depth, and lines and error bars represent the mean and standard error, respectively (Figure 2B). If you want to examine the unique or common features among samples or groups, the Venn diagram is a good way to show this pattern (Figure 3A). As for beta diversity, a heatmap based on Bray−Curtis dissimilarity would be a good visualization method. The colored grouping labels show how the samples cluster (Figure 2C).

## DISCUSSION

At present, for amplicon analysis, the most popular pipelines are QIIME [20] and QIIME 2 [15], which have been cited 54,900 times (Google Scholar, January 4, 2023). However, the two pipelines have some disadvantages that limit their use in microbiome analysis, such as a too‐large installation package, no support for the Windows system, and a lack of publication‐quality visualization. EasyAmplicon is trying to solve the above problems.

Currently, this is just the first version of EasyAmplicon. Runtime and memory usage depend on the data set size. The current version has been used by more than thousands of users and formally cited 36 times by the end of 2022 (searching “EasyAmplicon” in Google Scholar). The authors and core team of the WeChat account “Meta‐genome” will update the pipeline in time. The scripts for correlation, network analysis [38, 39], random forest [40, 41], machine learning [42], deep learning [43], transfer learning [44], and source track [45–47] analyses are ongoing and will be included in the pipeline soon. A webserver version like MicrobiomeAnalyst [48, 49] will be set up in the future. More general command‐line scripts and visualization styles are still in development, and they will be available in a new version of the pipeline. Anyone who is interested in this project is welcome to contribute scripts pertaining to analysis methods, visualization styles, and other issues mentioned in the GitHub repository.

## CONCLUSION

In summary, the EasyAmplicon pipeline provides an efficient, cross‐platform framework for amplicon analysis. Additionally, more than 20 predefined analysis and visualization solutions are provided for the multidimensional exploration of your data and for generating publication‐quality figures. Additionally, EasyAmplicon provides some utilities to be integrated with other widely used software for various needs.

## METHODS

### Quick start of EasyAmplicon

EasyAmplicon is coded mainly in Shell bash and R language and could be run in command‐line (terminal) mode or RStudio interactive mode. It is recommended to be deployed on a Windows system (with the installation of Git for Windows) and run in RStudio, especially for researchers without programming knowledge and skills. In addition, it also supports MacOS and Linux. To install it, please follow the instructions available at https://github.com/YongxinLiu/EasyAmplicon. Some dependent software and packages are listed in Table 1, and they are integrated for easy installation. The analysis process mainly includes three steps, as shown in Figure 1. To prove its practicability, we provide a demo data set that contains 18 samples belonging to three groups, and each sample is rarefied to 50,000 reads. This example data set is a subset of our previously published data (CRA001464) [50] (rarefied example data are deposited in GSA https://ngdc.cncb.ac.cn/gsa/, with accession ID: CRA002352).

### Dimensionality reduction (from sequences to tables)

The accepted input includes paired‐end or single‐end/merged sequences (fastq format), clean amplicons (fasta format), and even the intermediate files generated by other pipelines, as shown in Figure 1. Most amplicons are sequenced on the Illumina HiSeq. 2500 or NovaSeq. 6000 platform in paired‐end 250 bp mode. Typically, the pipeline starts with paired‐end reads in fastq format and merges them to get single‐end sequences. Primers and barcodes are cut, and then low‐quality reads are filtered out to get clean amplicons. These steps are performed mainly using vsearch [34] or USEARCH [19] (Figure 1A). The clean amplicons of 16S rDNA can be directly mapped to the reference database GreenGenes [51], and the closed‐reference operational taxonomic units (OTUs) table can be generated, which can be used as the input of PICRUSt to predict the potential functions [28, 52], and can be used as the input of BugBase for phenotypic prediction [30]. Alternatively, clean amplicons are usually clustered into OTUs (97% similarity) or denoised into amplicon sequence variants (ASVs) in de novo mode. Finally, the clean amplicons will be mapped to the de novo identified OTUs/ASVs to generate a feature table. Representative sequences can be used to construct a phylogenetic tree and perform taxonomic annotation (Figure 1A).

### Analysis (from big tables to small tables)

Feature tables are milestone outputs of the dimensionality reduction step. We can use the feature tables and phylogenetic tree to calculate all kinds of alpha‐ and beta‐diversity metrics. The feature tables with taxonomy annotations can be used to collapse into a specific taxonomic level and discover biomarkers at all taxonomic levels (Figure 1B). In addition, EasyAmplicon provides many glue scripts to generate input files for other widely used tools, such as QIIME 2 [15], STAMP [26], and LEfSe [27].

### Statistics and visualization (from tables to figures)

EasyAmplicon can generate visualizations for alpha diversity, beta diversity, taxonomic composition, and biomarkers along with related statistical tables (Figure 1C and 2; Table 2), and these publication‐quality graphs include a box‐plot, scatter plot, stacked bar plot, and heatmap. In addition, the output of EasyAmplicon can be imported into STAMP [26], or LEfSe [27] for biomarker identification, and visualized in an extended error bar plot or Cladogram, respectively.

**Table 2:** Summary of main visualization functions in EasyAmplicon

Plot	Script	Description	References
Boxplot	alpha_boxplot.R	The plot shows data distribution in each group. Dots represent each sample, and labeled letters indicate statistical significance among groups	[10]
Rarefaction curve	alpha_rare_curve.R	Richness of rarefied samples or groups from 1% to 100%	[10]
Venn diagram	sp_vennDiagram.sh	Visualizes common and unique elements among 2−5 groups	[53, 54]
Ordination scatter plot	beta_pcoa.R beta_cpcoa.R	Shows the results of dimensionality reduction	[55]
Heatmap	sp_pheatmap.sh compare_heatmap.sh	Shows distance or similarity matrix and deferential abundance features	[50, 56]
Stack plot	tax_stackplot.R	Shows taxonomic and functional composition in each sample or group	[57]
Cricular plot	tax_circlize.R	Shows taxonomic composition	[58]
Circle packing chart	tax_maptree.R	Shows the relations and relative abundance of taxonomic hierarchy	[59]
Volcano plot	compare_volcano.R	The dots in the plot show abundance and fold changes between two groups	[50]
Manhattan plot	compare_manhattan.sh	Shows taxonomy, abundance, and pattern between two groups	[60]

## AUTHOR CONTRIBUTIONS

Yong‐Xin Liu, Tao Wen, Tong Chen, Lei Chen, and Tengfei Ma developed the pipeline, performed the analysis, and wrote the manuscript. Xiaofang Li, Maosheng Zheng, Xin Zhou, Liang Chen, Xubo Qian, Jiao Xi, Hongye Lu, Huiluo Cao, Xiaoya Ma, Bian Bian, Pengfan Zhang, Jiqiu Wu, Ren‐You Gan, Baolei Jia, Linyang Sun, Zhicheng Ju, and Yunyun Gao have tested the pipeline, suggested pipeline amendments, and revised the manuscript. Tao Wen, Tong Chen, and Tengfei Ma write the codes for several visualization styles. Tong Chen improved the pipeline and designed the framework of visualization. Xin Zhou contributed to the VSEARCH analysis pipeline document. Yong‐Xin Liu, Tao Wen, Tong Chen, Xin Zhou, Xubo Qian, and Liang Chen contributed to the scripts and developed the user documentation. All authors have read the final manuscript and approved it for publication.

## CONFLICT OF INTEREST

The authors declare no conflict of interest.

## Supporting information

Supporting information.

## Data Availability

The raw sequence data reported in this paper have been deposited in the Genome Sequence Archive [61] in the National Genomics Data Center, China National Center for Bioinformation/Beijing Institute of Genomics, Chinese Academy of Sciences (GSA: CRA002352) that are publicly accessible at https://ngdc.cncb.ac.cn/gsa. EasyAmplicon is freely available and implemented in Shell and R, and easy to install. The step‐by‐step protocols can be found at GitHub https://github.com/YongxinLiu/EasyAmplicon or Gitee https://gitee.com/YongxinLiu/EasyAmplicon. All the data of the figures can be downloaded in GitHub, or supplementary tables.
